# Special Issue: Photocatalytic Materials for Environment Treatment and Energy Production

**DOI:** 10.3390/ma15103631

**Published:** 2022-05-19

**Authors:** Luminita Isac

**Affiliations:** Renewable Energy Systems and Recycling Research Center, Transilvania University of Brasov, Eroilor 29 Street, 35000 Brasov, Romania; isac.luminita@unitbv.ro

Nowadays, given the major problems facing humanity, the increasing environmental pollution and the need for sustainable and cheap energy sources represent important research issues. Therefore, the design and development of advanced materials that can be integrated into efficient products/technologies for environmental treatment and energy production are constant topics of research worldwide. In this context, photocatalytic materials are considered attractive candidates mainly for water treatment, but also for hydrogen production via photoelectrolytic water splitting. 

Photocatalytic technologies use light energy as a driving force to remove from (waste)water, through mineralization, persistent organic pollutants (e.g., dyes, pesticides and pharmaceuticals) in the presence of a photocatalytic material. There are a wide range of materials with photocatalytic activity, such as semiconductors (metal oxides, metal sulfides/selenides, etc.), semiconductor-based heterojunctions (micro/nano composite structures, binary or ternary hybrid structures, etc.), perovskites, transition metal spinel-type mixed oxides, metal organic frameworks (MOFs), hydrogels and waste-derived or templated materials

Thus, the topic of this issue mainly refers to the development of innovative, advanced and operational photocatalytic technologies that use new efficient, environmentally friendly, sustainable and reusable photocatalytic materials. The issue includes eight articles highlighting advanced photocatalytic materials with applications in both water treatment and hydrogen production through water splitting reaction.

Below is a brief summary of the papers included in this issue, considering the types of materials used in the photocatalytic process: metal oxide- (mono-, bi- or tricomponent hybrid structures), perovskite- and graphitic carbon nitride (g-C_3_N_4_)-based semiconductors.

Three of the total eight articles [1,2,3] are focused on TiO_2_-based photocatalysts, as TiO_2_ has been extensively studied as a low-cost and environmentally friendly material with relatively high photocatalytic activity and excellent chemical stability.

In Reference [1], TiO_2_ (Degussa P25) films were deposited using the doctor blade technique on three different substrates: microscopic glass (G), flour-doped tin oxide (FTO) and aluminum (Al). The photocatalytic properties of the samples were tested on two pollutants, tartrazine (Tr) dye and acetamiprid (Apd) insecticide, under UV-A, UV-B + C and VIS irradiations (seven scenarios) for 8 h. In order to optimize the photocatalysis efficiency, the influence of several parameters (irradiation source, total irradiance value, photon flux, catalyst substrate and pollutant type) were studied. The results showed that higher photocatalytic efficiencies were obtained for the sample obtained on conductive (Al) substrate (63.8% for Tr and 82.3% for Apd) using a mixture of three UV-A and one VIS sources (13.5 W/m^2^).

In Reference [2], the authors reported the obtaining of a novel Ba(II)/TiO_2_–MCM-41 composite using TiO_2_ doped with Ba^2+^ dispersed on MCM-41 molecular sieve. The enhanced photocatalytic efficiency of Ba(II)/TiO_2_–MCM-41 (91.7%) in the degradation of p-nitrobenzoic acid (4 × 10^−4^ M) under UV light irradiation (60 min) was assumed to the presence of Ba^2+^ ions and MCM-41, which contributed to the band gap energy lowering and to the facile dispersion of TiO_2_, resulting a mesoporous structure with a surface area of 341.2 m^2^/g. Moreover, the Ba(II)/TiO_2_–MCM-41 composite showed good recycling performance: after five consecutive cycles of reusability, the photocatalytic efficiency remained at a good value (73%).

One of the most serious threats to human health is the contamination of water with persistent organic pollutants loads from industrial, agricultural and transportation activities. Pesticides (herbicides, insecticides, fungicides) have high contamination potential in large agricultural areas and long-term persistence in the ecological system. In this context, the photocatalytic activity of dual S-scheme Cu_2_S_TiO_2__WO_3_ heterostructure, prepared by two-step sol–gel technique, in the degradation of S-Metholachlor (S-MCh) herbicide, under UV and UV–VIS irradiation light for 8h, was investigated in Reference [3]. It was reported that the three-component heterostructure shows higher photocatalytic efficiency (61%), compared with binary heterostructure (32% for Cu_2_S_TiO_2_) or mono-component structures (maximum 25%), due to the contribution of Cu_2_S component, with extended photo-response in VIS region of solar spectrum. 

References [4,5] are devoted to iron oxide-based photocatalysts. In Reference [4], the mineralogical characteristics, the band structure and photocatalytic properties of both natural goethite (FeOOH), obtained from Zhushan (China), and synthetic goethite samples are compared. The difference in the band gap values, 2.25 eV for natural goethite and 2.55 eV for its synthetic compound, is attributed to Al doping in natural goethite crystals, along with mineralogical tests. The photocatalytic experiments showed that natural goethite degrade methyl orange (MO, 25 ppm) with a higher efficiency (47.5%) than that of the synthetic goethite samples (31.5%). According to the authors, the theoretical simulation of band structure and photocatalytic properties of natural goethite are revealed for the first time in this paper; this contributes to our understanding of goethite-involved photochemical reactions in terrestrial environments.

Ferrite nanoparticles are another important class of narrow band gap materials with application in wastewater treatment, due to their high stability, strong magnetic properties, and high catalytic performances under VIS light irradiation. In Reference [5], the effect of Nd^3+^ ions substitution on the structural, optical and photo-Fenton activity of ZnNd_x_Fe_2−x_O_4_ ferrites nanoparticles (x = 0, 0.01, 0.03 and 0.05), prepared using a solution combustion technique, has been investigated. It was found that, with the addition of Nd^3+^ ions, the grain size and the optical bandgap energies decrease, thus showing a good photocatalytic activity. Indeed, the photocatalytic experiments results indicated that the ZnNd_x_Fe_2−x_O_4_ samples show higher removal efficiencies than the pure ferrites (ZnFe_2_O_4_) in the degradation of Rhodamine B (RhB, 10 mg/L) with H_2_O_2_ (0.04 mM) addition, under VIS light irradiation. The highest degradation efficiency (98%) was obtained after 210 min using H_2_O_2_ and ZnNd_0.03_Fe_1.97_O_4_ (0.75 g/L) as photocatalyst. Without the addition of H_2_O_2_ in dye solution, the degradation efficiency was only 28% in the same conditions. The enhanced photocatalytic activity of the Nd^3+^ doped ferrite is the result of the efficient separation and migration of photoinduced electron and holes. 

Perovskite oxides, ceramic materials with the general formula ABO_3_, are recognized as efficient catalysts in the degradation of several organic pollutants from water, due to their stable and flexible structure that allows a lot of variety of properties. Among perovskite oxides, strontium titanate (SrTiO_3_) with cubic structure exhibit photocatalytic activity under UV light. According to Reference [6], Bi-doped SrTiO_3_ perovskites (Sr_1−x_Bi_x_TiO_3_, x = 0, 0.03, 0.05, 0.07, 0.1) were prepared via a solid-state method and tested as photocatalysts in the degradation of the azo-dye acid orange 7 (AO7, 10 mg/ L) under VIS light irradiation for 1 h. The enhanced photocatalytic activity of all doped samples (67.8% for Sr_0.95_Bi_0.05_TiO_3_), compared with undoped SrTiO_3_ (3.7%), is more probably due the heterostructure formation, with valence and conduction bands distributed such that the recombination of photogenerated electron–hole pair is reduced. Reusability and ecotoxicity evaluation (D. magna) tests indicate that Sr_0.95_Bi_0.05_TiO_3_ may act as a visible-light-driven photocatalyst to degrade both the AO7 dye as well as its toxic by-products. 

In Reference [7], graphitic carbon nitride (CN), a metal-free semiconducting material with good mechanical, chemical and thermal stability and a band gap of 2.7 eV (absorption in VIS region), was prepared by the heating of melamine (CN-M, Eg = 2.64 eV, SSA = 12 m^2^/g) and melamine-cyanurate complex (CN-MCA, Eg = 2.73 eV, SSA = 225 m^2^/g), respectively, in air at 550 °C for 4 h. The photocatalytic activity of the CN materials (45 mg/150 mL pollutant solution) was tested for the decomposition of antibiotics ofloxacin and ampicillin (20 mg/L) under VIS light irradiation. The results shows that ofloxacin was decomposed more efficiently than ampicillin, the photocatalytic activity of CN-MCA being higher (90% in 1 h) for ofloxacin degradation, compared with CN-M (80% in 2 h), due to its higher SSA and lower Eg.

The authors of Reference [8] demonstrate that the introduction of noble metals (Pt, Ir, Ru) on the carbon nitride surface, using a wet impregnation method with an excess of solvent, has as a result a significant increase in g-C_3_N_4_ photocatalytic activity during water splitting under visible light. The reduction of the photocatalysts with hydrogen generated several structural changes (e.g., an increase in the specific surface area, in the C/N atomic ratio and number of defects in the crystalline structure of g-C_3_N_4_), which significantly enhanced the photocatalytic activity of metal/g-C_3_N_4_ catalysts due to better separation of photogenerated electron–hole pairs on metal nanoparticles. It was reported that the activity of the Pt/g-C_3_N_4_ photocatalyst was 24 times higher than that of pristine (g-C3N4).
